# AmiCa: Atlas of miRNA-gene correlations in cancer

**DOI:** 10.1016/j.csbj.2024.05.030

**Published:** 2024-05-21

**Authors:** Nina Hauptman, Jože Pižem, Daša Jevšinek Skok

**Affiliations:** a Institute of Pathology, Faculty of Medicine, University of Ljubljana, Slovenia; b Agricultural Institute of Slovenia, Slovenia

**Keywords:** miRNA, Gene, Expression, Cancer, Correlation, Gene prioritization

## Abstract

The increasing availability of RNA sequencing data has opened up numerous opportunities to analyze various RNA interactions, including microRNA-target interactions (MTIs). In response to the necessity for a specialized tool to study MTIs in cancer and normal tissues, we developed AmiCa (https://amica.omics.si/), a web server designed for comprehensive analysis of mature microRNA (miRNA) and gene expression in 32 cancer types. Data from 9498 tumor samples and 626 normal samples from The Cancer Genome Atlas were obtained through the Genomic Data Commons and used to calculate differential expression and miRNA-target gene (MTI) correlations. AmiCa provides data on differential expression of miRNAs/genes for cancers for which normal tissue samples were available. In addition, the server calculates and presents correlations separately for tumor and normal samples for cancers for which normal samples are available. Furthermore, it enables the exploration of miRNA/gene expression in all cancer types with different miRNA/gene expression. In addition, AmiCa includes a ranking system for genes and miRNAs that can be used to identify those that are particularly highly expressed in certain cancers compared to other cancers, facilitating targeted and cancer-specific research. Finally, the functionality of AmiCa is illustrated by two case studies.

## Graphical Abstract

**Figure:**
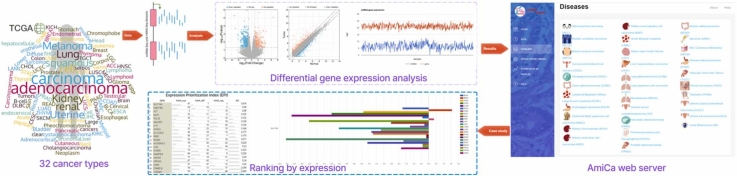

## 1 Introduction

Today, cancer remains one of the leading causes of death worldwide and presents a significant challenge for the healthcare system [1]. To uncover the critical genomic and genetic changes in cancer, it is essential to explore various aspects, including genomic alterations, methylation patterns, and changes in mRNA and microRNA (miRNA) expression levels. MiRNAs play a crucial role in carcinogenesis by binding to mRNAs and consequently influencing the protein profile of the cell [2]. The quantification of miRNA and gene levels has advanced in recent years with new high-throughput technologies such as RNA sequencing (RNA-Seq) [3], [4]. Although the method provides transcriptomic data of immense importance for the development of novel disease therapies, the time-consuming and complex data analysis tends to limit its translational potential.

As a result, there are several platforms and databases that are widely used in this field to collect, analyze, and visualize genomic data in the context of cancer research, such as GDC [5], cBioPortal [6], XENA [7], miRBase [8], miRTarBase [9], GEPIA2 [10], Tissue Atlas [11], FIREBROWSER (http://firebrowse.org/), dbDEMC [12], IMOTA [13], Cancer miRNA census [14]. One of the largest sources of experimental cancer data is The Cancer Genome Atlas (TCGA), hosted on the GDC portal, which provides genomic data for numerous tumor and normal samples across various cancer types. This includes gene expression data, miRNA expression data, and clinical information, enabling researchers to explore gene expression and miRNA-target gene correlations in cancer on a large scale. Although there are alternative big data sources available, data obtained from different databases cannot be directly compared [15]. Detecting miRNA/gene correlations may contribute to the development of new prognostic, diagnostic and therapeutic solutions in cancer. With the development of miRNA target gene identification methods, tools for exploring the interactions between miRNAs and their target genes, miRNA-target interactions (MTIs), have also been established, including miRTarBase, which contains MTI correlations [9]. However, the correlations in miRTarBase are only presented for patients with paired tumor and normal tissues. To provide an alternative that includes correlations of all miRNAs and mRNAs from RNA-Seq data available for both cancer and normal tissues, we developed a platform with extended comprehensive potential. The correlations between miRNA-mRNA levels are calculated not only for tumor samples but also for normal samples. Depending on the research interest, the user can search for correlations of MTIs in either tumor or normal tissues or investigate how the correlations are perturbed when cancer occurs. The AmiCa platform offers various options, from searching expression profiles of miRNAs and genes to exploring MTIs, available for individual cancer or across cancer types. Users can even match miRNA and gene expression for pairs that are not confirmed MTIs. Additionally, our tool allows users to rank miRNAs or genes based on selected cancer compared to other cancers in the database. The platform also delivers user-friendly query results through meaningful graphs. Our primary objective with AmiCa is to offer an openly accessible interactive catalog of MTIs alongside their expression data, fostering improved target identification methods. Notably, our aim is to facilitate quicker and a more accessible utilization of valuable gene expression data.

## 2 Materials and methods

### 2.1 Data curation

We retrieved miRNA and mRNA expression data for all TCGA projects from the GDC portal [5] using the TCGAbiolinks package in R [16].

Included mRNA data was previously normalized using the FPKM-UQ method (Fragments Per Kilobase of transcript per Million mapped reads Upper Quartile). The formalin-fixed paraffin-embedded samples were excluded from the dataset because multiple aliquots were sequenced, resulting in duplicated entries in the mRNA data. After excluding these samples, our mRNA dataset contained 9785 tumor samples and 730 normal samples.

Similarly, the miRNA data, was pre-mapped and normalized by RPM (Reads Per Million miRNA mapped). The miRNA data has information on pre-miRNA IDs, isoform coordinates, read counts, RPM and miRNA regions. The miRNA region provides two information, one specifies the nature of the transcript, such as mature, stem-loop, precursor, or an unannotated read, and the other provides miRNA MIMAT ID, which identifies specific mature miRNAs in miRBase [8]. Only mature miRNAs were retained, for which RPM values were aggregated. Therefore, we obtained a new matrix with aggregated RPM values for each mature miRNA per sample. The annotation of miRNAs was performed using miRBase (version 22.1) [8]. By aggregating mature miRNA regardless of isoform coordinates, we obtained mixed isomiRs of mature miRNAs [17]. The miRNA dataset contained no formalin-fixed paraffin-embedded samples; therefore, we retained all 9879 tumor and 675 normal samples.

To perform correlation analysis of MTIs, it was necessary to use samples with paired miRNA/gene data. Consequently, this intersect consisted of 9498 tumor and 626 normal samples. The samples belonged to 33 cancer types, but the intersection between miRNA and mRNA data included only 32 cancer types. In glioblastoma (GBM), there were no tumor samples with paired miRNA/gene data; however, there were five normal samples in GBM with such paired data. Therefore, these five samples were combined with the Lower Grade Glioma (LGG) dataset, as both projects include samples derived from brain tissue.

### 2.2 Analysis of gene and miRNA expression in cancer

To determine the regulatory impact of miRNAs/genes on specific cancers, we assessed their differential expression.

In the mRNA dataset, we first averaged the FPKM-UQ values across tumor samples for each cancer type and then applied a log_2_ transformation. The same was applied for the normal samples from the same TCGA project. As a result, we obtained log_2_(average FPKM-UQ) for each group of samples. To obtain the log_2_ fold change value (log_2_FC), we subtracted the log_2_-transformed average FPKM-UQ value of normal samples from that of tumor samples for each project separately.

The same procedure was applied in the miRNA dataset on RPM values obtaining log_2_(average RPM) for each sample group and log_2_FC for miRNA.

These miRNA/gene log_2_FC values are displayed on the first page of each cancer that includes normal samples, including bladder urothelial carcinoma (BLCA), breast invasive carcinoma (BRCA), cervical and endocervical cancers (CESC), cholangiocarcinoma (CHOL), colon adenocarcinoma (COAD), esophageal carcinoma (ESCA), head and neck squamous cell carcinoma (HNSC), kidney chromophobe (KICH), kidney renal clear cell carcinoma (KIRC), kidney renal papillary cell carcinoma (KIRP), brain lower grade glioma (LGG), liver hepatocellular carcinoma (LIHC), lung adenocarcinoma (LUAD), lung squamous cell carcinoma (LUSC), pancreatic adenocarcinoma (PAAD), pheochromocytoma and paraganglioma (PCPG), prostate adenocarcinoma (PRAD), rectum adenocarcinoma (READ), skin cutaneous melanoma (SKCM), stomach adenocarcinoma (STAD), thyroid carcinoma (THCA), thymoma (THYM) and uterine corpus endometrial carcinoma (UCEC). Furthermore, these values were used in all sections of the “Expression by disease", while log_2_(average FPKM-UQ) and log_2_(average RPM) are displayed in the MTI reports.

For cancers without normal samples, such as adrenocortical carcinoma (ACC), lymphoid neoplasm diffuse large B-cell lymphoma (DLBC), acute myeloid leukemia (LAML), mesothelioma (MESO), ovarian serous cystadenocarcinoma (OV), sarcoma (SARC), testicular germ cell tumors (TGCT), uterine carcinosarcoma (UCS) and uveal melanoma (UVM) the log_2_FC was undeterminable. For these cancers the first page of the cancer shows log_2_(average FPKM-UQ) for gene and log_2_(average RPM) for miRNA. Due to lack of normal samples, these cancers were not included in sections of the “Expression by disease”: “miRNA/gene expression”, “Genes Ranked by Expression” and “miRNA ranked by Expression”.

### 2.3 Target genes

The validated list of MTIs obtained from miRTarBase included 381,833 MTIs [9]. These MTI pairs consisted of various combinations of 2451 miRNAs and 19,587 target genes. The miRNAs/genes that were part of these MTIs were intersected with miRNA/gene data from TCGA, resulting in 316,056 MTIs, where both the miRNA and gene were expressed in at least ten samples of any given cancer.

### 2.4 Correlations between miRNA and their target genes

Correlation analyses were performed on samples with paired miRNA/gene data. Prior analysis, we transformed the data using log_2_(FPKM-UQ+1) for target genes and log_2_(RPM+1) for miRNAs. The correlation coefficient (R) was calculated for 316,056 MTIs using the Pearson correlation test to assess the relationship between the expressed miRNAs and their target genes. Correlation coefficient R was calculated separately for tumor and normal samples in each cancer, except in datasets lacking normal samples, where correlations were only calculated for tumor samples. Correlations were determined using the R package "sigr" [18] when more than three samples were available; otherwise, correlation calculations were not feasible.

### 2.5 Platform design

The data included in our study is stored in a relational MySQL database management system (http://www.mysql.com). A web interface designed with HTML, CSS and JavaScript and hosted on an Apache web server facilitates data retrieval. Interactive charts were created with Google Chart Tools (https://developers.google.com/chart/) using a combination of PHP scripts and MySQL queries to manipulate the data.

### 2.6 Ranking miRNA/gene expression

We developed an Expression Prioritization Index (EPI) to rank miRNA/gene expression by priority. The EPI is calculated as the sum of products between the relative weights (w_i_) and the ranks of the i-th criteria. We considered three criteria: 1) the most overexpressed miRNA/gene in the studied cancer (w = 0.1), 2) the difference to the second most expressed miRNA/gene (w = 0.3), and 3) the number of underexpressed genes/miRNAs (w = 0.6).

Firstly, we categorized genes based on three criteria: gene expression (rang_exp), differential gene expression (rang_diff), and the count of cancers where the gene exhibits negative expression (rang_neg). We then transformed these values, which originally ranged from 1 to 100, into decimal fractions, referred to as "rang decimal fractions" (RDF).

RDF = rang / 100

Subsequently, we calculated the EPI for genes and miRNAs in eight cancers using the formula:

EPI = (rang_exp * 0·1) + (rang_diff * 0·3) + (rang_neg * 0·6)

**Fig. 1:**
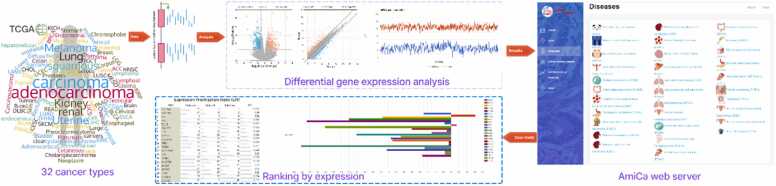
An illustration of the AmiCa workflow. miRNA and gene expression data were extracted from The Cancer Genome Atlas (TCGA) and used to calculate differential expression and miRNA-target genes (MTIs) correlations. The resulting expression and correlation values are visualized on the AmiCa web platform. Finally, AmiCa generates tables and graphs that can be easily downloaded for further analyzes, such as prioritizing miRNAs and genes by ranking.

Fig. 1 presents a graphical abstract that illustrates the implementation of the AmiCa platform. The workflow details the sequence of events, including data extraction and processing, tool development, results visualization, and tool applicability.

## 3 Results

### 3.1 AmiCa platform overview

**Fig. 2:**
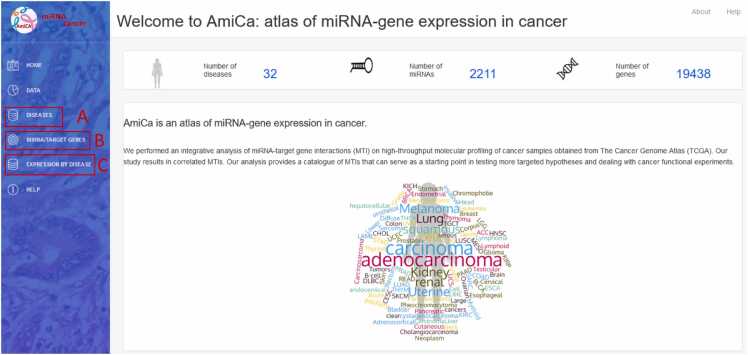
First page of the AmiCa platform. Users can study miRNA and gene expressions and their correlations in each of the 32 cancers (A), explore miRNA/target gene interactions across cancers (B), and compare and rank miRNA and/or gene expressions across cancers via tabular and graphical representations (C).

The AmiCa platform enables expression studies to be carried out in a variety of ways. Fig. 2 shows the homepage of the AmiCa platform. It illustrates how users can navigate through the various analysis options, including examining miRNA and gene expression and their correlations within individual cancer types (Fig. 2A), expression and correlation of MTIs across different cancer types (Fig. 2B), and expression of miRNAs and genes across different cancer types, as well as ranking genes and miRNAs based on their expression relative to other cancer types (Fig. 2C).

### 3.2 Diseases

The dropdown menu “Diseases” in the sidebar enables selection from 32 different types of cancer (Fig. 2A). The first page for each cancer (Fig. 4) displays data on patients and samples included in the analysis, such as statistics by gender, disease type, and race (Fig. 4A). It features a graphical view of the ten genes and miRNAs that are most up/downregulated in cancers with normal tissue samples; for cancers lacking normal samples, the view displays the ten most expressed miRNAs/genes in tumor samples (Fig. 4B). The first page also contains search forms for entering a miRNA or gene name (Fig. 4C).

**Fig. 3:**
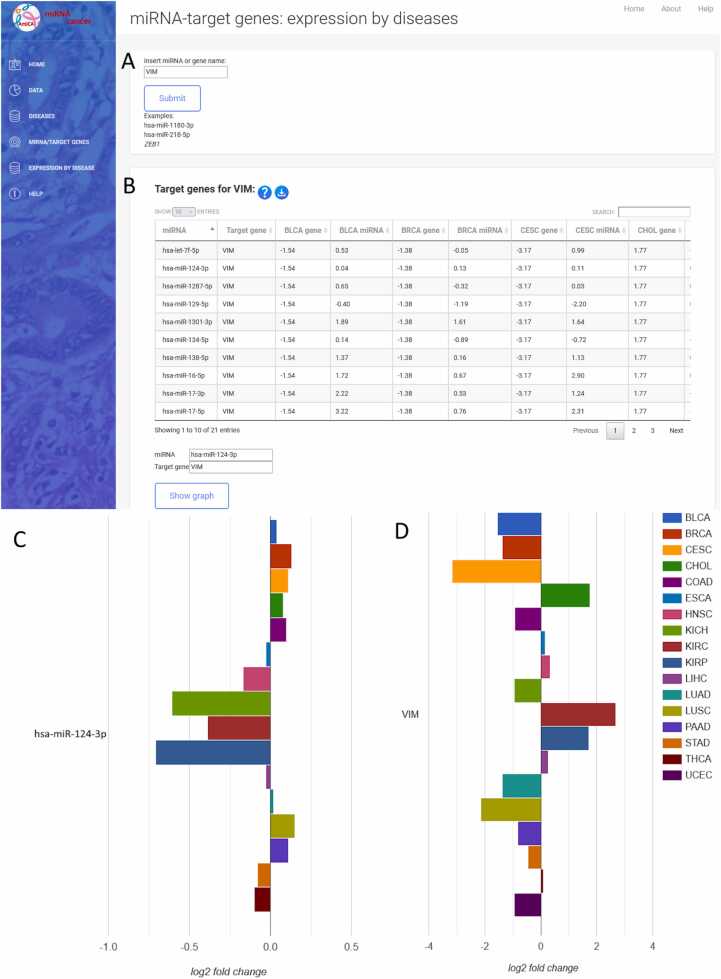
Expression by diseases of the selected miRNA/gene. A) query input for miRNA/gene; B) results presented in table format with the differential expression across different cancers; C) graphic presentation of selected miRNA and D) gene across cancers with normal tissue samples.

**Fig. 4:**
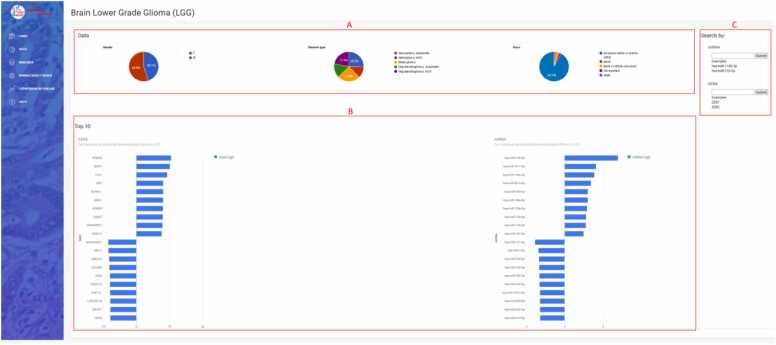
Welcome page to study miRNA and gene expression in brain lower grade glioma (LGG). (A) statistics by gender, disease type, and race; (B) graphical view of the ten genes and miRNAs that are most upregulated and/or downregulated in LGG; (C) search forms for entering the miRNA or gene name for further study of their expression in LGG.

In the miRNA form, the miRNA name (e.g., hsa-miR-124–3p) or a part of the miRNA name (e.g., 124) can be entered and miRNA selected from a dropdown list. The form “Search by gene” has the same function in selecting desired genes. The dropdown lists contain the union of all miRNAs and genes expressed in at least one cancer type but are not necessarily represented in every cancer type. Upon selecting a miRNA/gene of interest, a report consisting of four parts is generated and opens in a new tab. The summary section includes the number of tumor and normal (when available) samples with paired miRNA/gene data, the log_2_(average RPM) for the selected miRNA or the log_2_(average FPKM-UQ) for the selected gene for tumor and normal samples (when available). The second part of the report displays distribution of miRNA/gene expression in the analyzed tumor and normal (when available) tissue samples. The third part shows the log_2_FC values for the ten most upregulated and ten most downregulated target genes (if there are ten or more target genes) for miRNA searches. For gene searches, it shows the most up/downregulated miRNAs for the selected target gene. If normal samples are not available, top the most expressed miRNAs/genes are displayed. The last part displays a table of all known MTIs for the searched miRNAs/genes. This table allows searches and sorting of individual columns by clicking the arrows at the end of each column. Clicking on a row automatically fills in the form at the bottom of the page with the names of the miRNA and gene. The selection is then transmitted via the "Show graph" button.

**Fig. 5:**
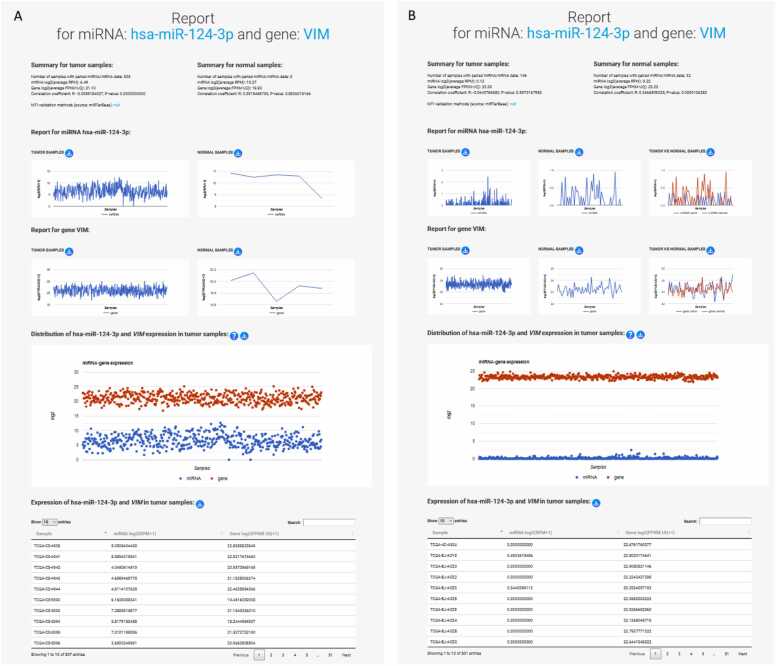
Utilization of miRNA/gene expressions and their correlation within a specific cancer. Report for miRNA hsa-miR-124–3p and its target gene *VIM* for brain lower grade glioma (LGG) (A) and thyroid carcinoma (THCA) (B).

A new tab opens a four-part report for the specific (previously selected) MTI (Fig. 5), where the MTIs and their correlations within each cancer are examined in more detail. The expression of miRNA/gene on these pages is represented by log_2_-transformed values of miRNA/gene expressions for individual samples, which are used for graphing expression values and calculating the correlation of the MTIs. Each MTI report provides a detailed summary for both tumor and normal samples, including the number of samples with paired miRNA/gene data that also had a non-zero expression value, necessary for the R calculation. The summary includes the log_2_(average RPM) for the miRNA and the log_2_(average FPKM-UQ) for the gene, the calculated R and p-value for the selected MTI, and the MTI validation methods from miRTarBase. Notably, the sample size may sometimes be smaller than the entire dataset, due to R calculation is performed for samples with non-zero values for miRNA/gene data. The second part contains graphs showing miRNA and gene expression in tumor and normal samples, and, where applicable, a comparison between expressions in tumor and normal for paired tumor and normal samples. The third part of the MTI report shows the expression in tumor tissue by sample, presented as a distribution in a graphical and tabular view.

### 3.3 miRNA/target genes

The miRNA/target gene section (Fig. 2B) focuses on the graphical presentation of the differential expression of MTIs across different cancer types with visualization option. Only cancers with normal samples are included in this section, as the difference in expression between tumor and normal samples is crucial for the comparison of different tissue types.

The miRNA/gene name is entered in the textbox (Fig. 3A). Upon clicking the search button, a table displays the differentially expressed miRNAs/genes across various cancers (Fig. 3B). Selecting a row triggers an autofill query beneath the table, accompanied by a "Show Graph" button. This button generates a graph that visually presents differential expression of the selected miRNA (Fig. 3C) and gene (Fig. 3D) across multiple other cancers.

### 3.4 Expression by disease

Selecting the “Expression by Disease” dropdown menu in the sidebar opens three applications to examine genes and miRNAs expressed in cancer types, where normal tissue samples were available, namely: miRNA/gene expression, genes ranked by expression, and miRNA ranked by expression (Fig. 2C). The result is available in a tabular and graphical form. The miRNA/gene expression application opens two search forms in which the miRNA and/or the gene name (not necessarily MTI) can be entered. The results are displayed as two bar charts for the searched miRNA/gene with differential expression values in different cancer types.

Gene and miRNA ranking based on their differential expression, compared to other cancer types, is available by selecting “Genes ranked by expression” and “miRNA ranked by expression” from the dropdown menu. Using the radio buttons on the left side of the website, a specific cancer type can be selected (Fig. 7). The result is displayed in the table with an export option. To search for specific miRNA/gene, a search box above the table on the right can be used. Clicking on the row or name of the miRNA/gene, the preset search window is filled, which draws a bar chart with the differential expression values of this miRNA/gene in cancer types, where normal tissue samples were available. The chart is also easily exported.

The rank displayed in the table represents the number of cancers in which the expression of the miRNA/gene is lower than that of the selected cancer (up to a maximum of 22).

### 3.5 Case study 1

#### 3.5 .1. miRNA/gene expressions and their correlations in case of brain lower grade glioma

AmiCa provides multiple ways to study miRNA/gene expression in the cancer of interest. Below we demonstrate the applicability of the AmiCa platform on a practical example of one cancer type, brain lower grade glioma (LGG). First, we selected LGG among other cancer types through the “Diseases” tab in the sidebar menu (page not shown).

Statistics of the data used in the LGG study showed that the analysis was performed on 507 samples (54.9 % male, 45.1 % female) and that the majority of included patients were white (92.1 %). Diagnoses were classified into five sub-diagnoses: anaplastic astrocytoma, not otherwise specified (NOS) astrocytoma, mixed glioma, anaplastic oligodendroglioma, and oligodendroglioma, NOS. The predominant diagnoses were anaplastic astrocytoma and mixed glioma with 128 and 126 samples, respectively (Fig. 4A). Gene *HOXD8* and miRNA hsa-miR-10b–5p were found to be the most up-regulated in LGG, while gene *AC006538.2* and miRNA hsa-miR-137–3p were the most down-regulated in LGG (Fig. 4B).

AmiCa offers users the ability to conduct MTI searches based on specific genes or miRNAs. When focusing on the gene *VIM*, our analysis revealed 24 validated MTIs with various miRNAs. Among these interactions, the most significant negative correlation emerged for hsa-miR-124–3p (Fig. 5A).

Gene and miRNA expression in LGG was calculated based on 507 tumor and five normal samples (from the GBM dataset). These five normal samples were not paired with tumor samples, as it is common in other cancers. Therefore, as an example of expression with paired tumor and normal samples, we included a report for the selected MTI in the case of THCA (Fig. 5B).

#### 3.5.2 Expression by disease

##### 3.5 .2.1. miRNA/gene expression

**Fig. 6:**
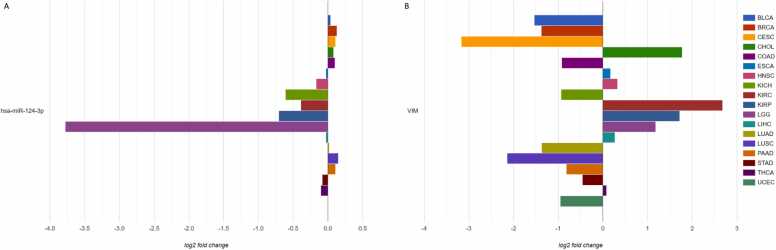
Expression of miRNA hsa-miR-124–3p (A) and gene *VIM* (B) by each included cancer.

To study the expression of a specific miRNA and gene in LGG, we selected miRNA hsa-miR-124–3p (Fig. 6A) and gene *VIM* (Fig. 6B). Notably, AmiCa enables the user to choose a specific miRNA-gene pair that is not a confirmed MTI, so any miRNA-gene combination can be presented, regardless. The results show that hsa-miR-124–3p is the most downregulated miRNA in LGG with log_2_FC –3.78, while the gene *VIM* is upregulated in LGG with log_2_FC 1.18. Fig. 6B also shows that the highest expression of *VIM* is present in KIRC.

##### 3.5 .2.2 Genes and miRNAs ranked by expression

**Fig. 7:**
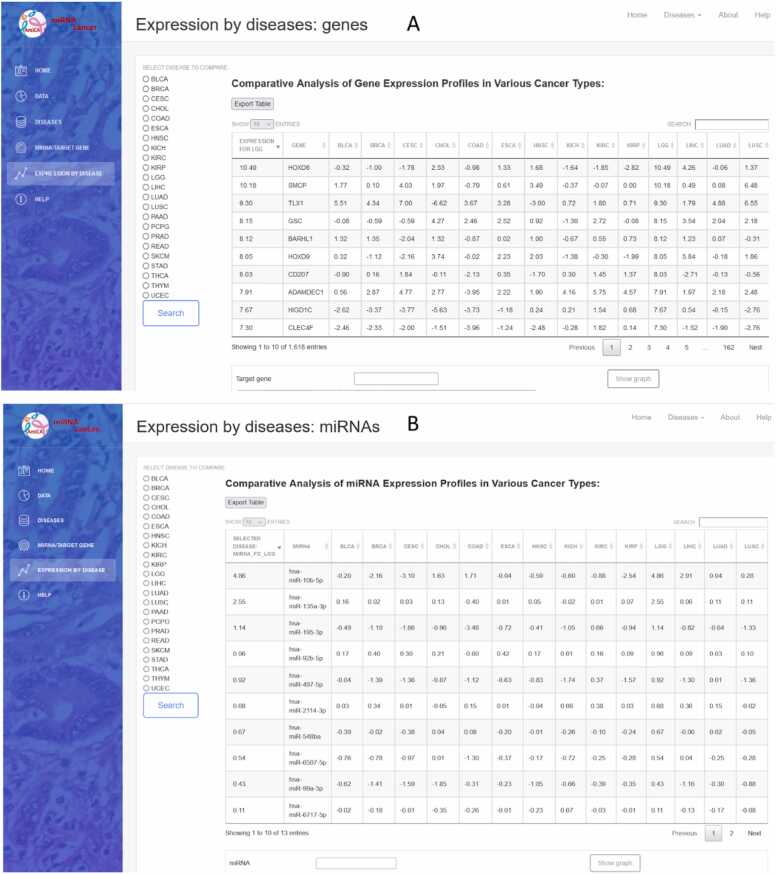
Gene and miRNA ranking according to their expression profiles in the case of brain lower grade glioma (LGG). (A) gene expression ranking by disease and (B) miRNA expression ranking by disease.

By selecting LGG using the radio buttons on the left side of the website (Fig. 7), a comparative analysis of differential gene expression profiles in different cancer types revealed 1618 genes with the highest rank (Fig. 7A). The *HOXD8* gene appeared as the most highly expressed gene in LGG with a log_2_FC of 10.49, followed by its second highest expression in LIHC with a log_2_FC of 4.26. The gene was downregulated in as many as 12 cancer types, with the lowest expression in KIRP.

A comparative analysis of miRNA expression in LGG versus other cancer types showed 13 miRNAs with a rank of 22. The most highly expressed miRNA was hsa-miR-10b–5p with a log_2_FC of 4.86 in LGG samples, while in LIHC and READ the log_2_FC was 2.91. This miRNA was downregulated in 13 cancer types, with the lowest expression in CESC.

### 3.6 Case study 2

In the second case study, we employed gene and miRNA rankings based on their expression to illustrate the utility of AmiCa. We focused on identifying specific genetic markers capable of distinguishing primary liver adenocarcinomas from those with metastatic potential to the liver.

For the eight cancer types comprising BRCA, CHOL, COAD, LIHC, LUAD, PAAD, READ and STAD, we obtained a list of the most overexpressed genes/miRNAs from the "expression by disease" section, which was crucial in the development of the Expression Prioritization Index (EPI).

The top genes/miRNAs for each of these eight cancers were extracted from the AmiCa platform.

For gene prioritization, our analysis starting point was the initial 100 genes identified as the most overexpressed in the "expression by disease" section, under the "Genes Ranked by Expression" subsection. Table 1 outlines the top genes in each category, forming the basis for constructing the EPI, and includes the addition of the EPI's most prioritized gene. The complete gene lists with EPI rank are included in Supplementary Table 1.

**Table 1:** Top prioritized genes in each category that composes EPI for each selected cancer.

**Cancer**	**Gene Expression** **rank no. 1**	**Differential gene expression** **rank no. 1**	**Count of negative gene expression** **rank no. 1**	**Expression prioritization index** **rank no. 1**
**BRCA**	*MS4A15*	*SCT*	*SLC17A8*	*SLC17A8*
**CHOL**	*CST1*	*C2orf50*	*SLC5A8*	*WIF1*
**COAD**	*SFTA2*	*DKK4*	*SLC22A3*	*MUC6*
**LIHC**	*MAGEA1*	*GPC3*	*GPC3*	*GPC3*
**LUAD**	*CLPSL2*	*SPP2*	*ETNPPL*	*ETNPPL*
**PAAD**	*UGT1A10*	*SI*	*TMEM238*	*CIDEC*
**READ**	*C6orf15*	*BHLHA9*	*GRIN2*	*AL662899.3*
**STAD**	*CST4*	*R3HDML*	*HJV*	*C4BPA*

EPI, expression prioritization index; BRCA, breast invasive carcinoma; CHOL, cholangiocarcinoma; COAD, colon adenocarcinoma, LIHC, hepatocellular carcinoma; LUAD, lung adenocarcinoma; PAAD, pancreas adenocarcinoma, READ, rectum adenocarcinoma; STAD, stomach adenocarcinoma.

**Fig. 8:**
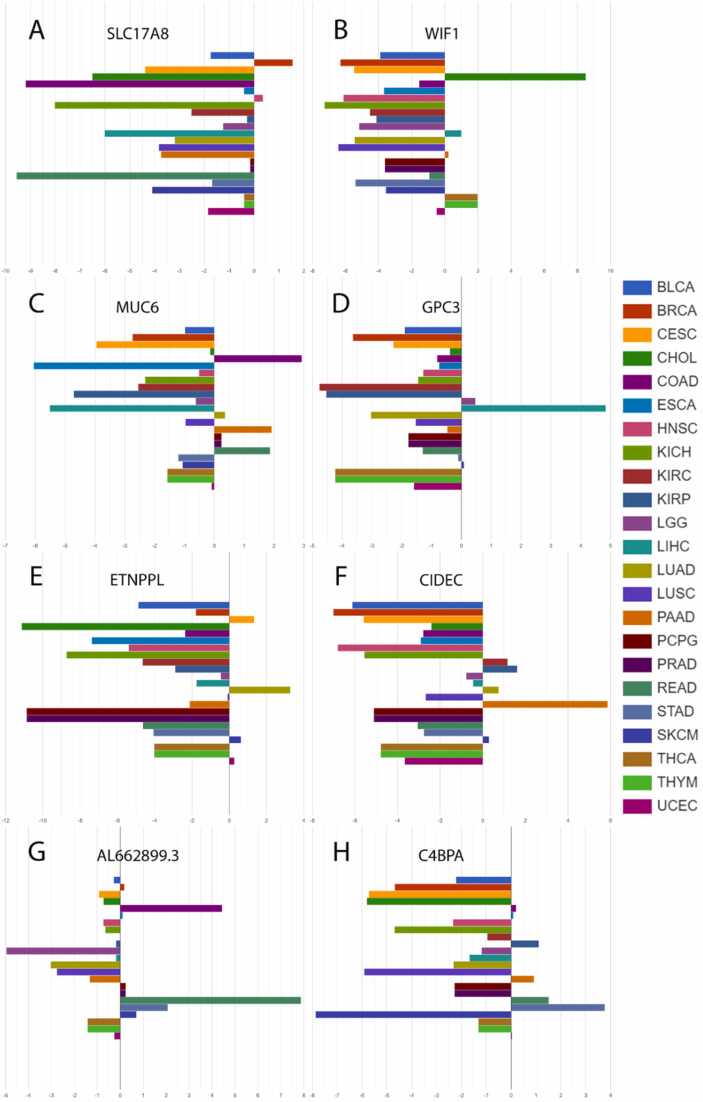
First prioritized genes according to Expression Prioritization Index (EPI) of (A) breast invasive carcinoma (BRCA), (B) cholangiocarcinoma (CHOL), (C) colon adenocarcinoma (COAD), (D) hepatocellular carcinoma (LIHC), (E) lung adenocarcinoma (LUAD), (F) pancreas adenocarcinoma (PAAD), (G) rectum adenocarcinoma (READ) and (H) stomach adenocarcinoma (STAD).

Using the AmiCa's "expression by disease" section (Fig. 8), the first-prioritized genes for each cancer were drawn. The first prioritized genes according to EPI were: *SLC17A8* in BRCA, *WIF1* in CHOL, *MUC6* in COAD, *GPC3* in LIHC, *ETNPPL* in LUAD, *CIDEC* in PAAD, *AL662899.3* in READ and *C4BPA* in STAD.

For miRNA analysis, data obtained by AmiCa often yielded less than 100 miRNAs due to data limitation, with varying counts for each cancer type (e.g., BRCA: 7 miRNAs, CHOL: 14 miRNAs, COAD: 57 miRNAs, LIHC: 10 miRNAs, LUAD: 23 miRNAs, PAAD: 12 miRNAs, READ: 193 miRNAs, and STAD: 7 miRNAs). These results were extracted from the "expression by disease" section under the "miRNAs Ranked by Expression" subsection. Table 2 presents the top miRNAs in each category, forming the foundation for constructing the EPI, and includes the addition of the EPI's most prioritized miRNA. Detailed lists of miRNAs along with their EPI rank are available in Supplementary Table 2.

**Table 2:** Top prioritized miRNAs in each category that composes EPI for each selected cancer.

**Cancer**	**Gene Expression** **rank no. 1**	**Differential gene expression** **rank no. 1**	**Count of negative gene expression** **rank no. 1**	**Expression prioritization index** **rank no. 1**
**BRCA**	*hsa-miR-184*	*hsa-miR-184*	*hsa-miR-184*	*hsa-miR-184*
**CHOL**	*hsa-miR-92b-3p*	*hsa-miR-133a-3p*	*hsa-miR-133a-3p*	*hsa-miR-133a-3p*
**COAD**	*hsa-miR-374a-3p*	*hsa-miR-145-3p*	*hsa-miR-582-3p*	*hsa-miR-143-3p*
**LIHC**	*hsa-miR-216b-5p*	*hsa-miR-216b-5p*	*hsa-miR-216a-3p*	*hsa-miR-216b-5p*
**LUAD**	*hsa-miR-3607-3p*	*hsa-miR-154-5p*	*hsa-miR-143-5p*	*hsa-miR-143-5p*
**PAAD**	*hsa-miR-1247-5p*	*hsa-miR-125b-2-3p*	*hsa-miR-100-5p*	*hsa-miR-100-5p*
**READ**	*hsa-miR-552-5p*	*hsa-miR-26a-5p*	*hsa-miR-552-5p*	*hsa-miR-126-3p*
**STAD**	*hsa-miR-194-3p*	*hsa-miR-654-5p*	*hsa-miR-194-3p*	*hsa-miR-194-3p*

EPI, expression prioritization index; BRCA, breast invasive carcinoma; CHOL, cholangiocarcinoma; COAD, colon adenocarcinoma, LIHC, hepatocellular carcinoma; LUAD, lung adenocarcinoma; PAAD, pancreas adenocarcinoma, READ, rectum adenocarcinoma; STAD, stomach adenocarcinoma.

**Fig. 9:**
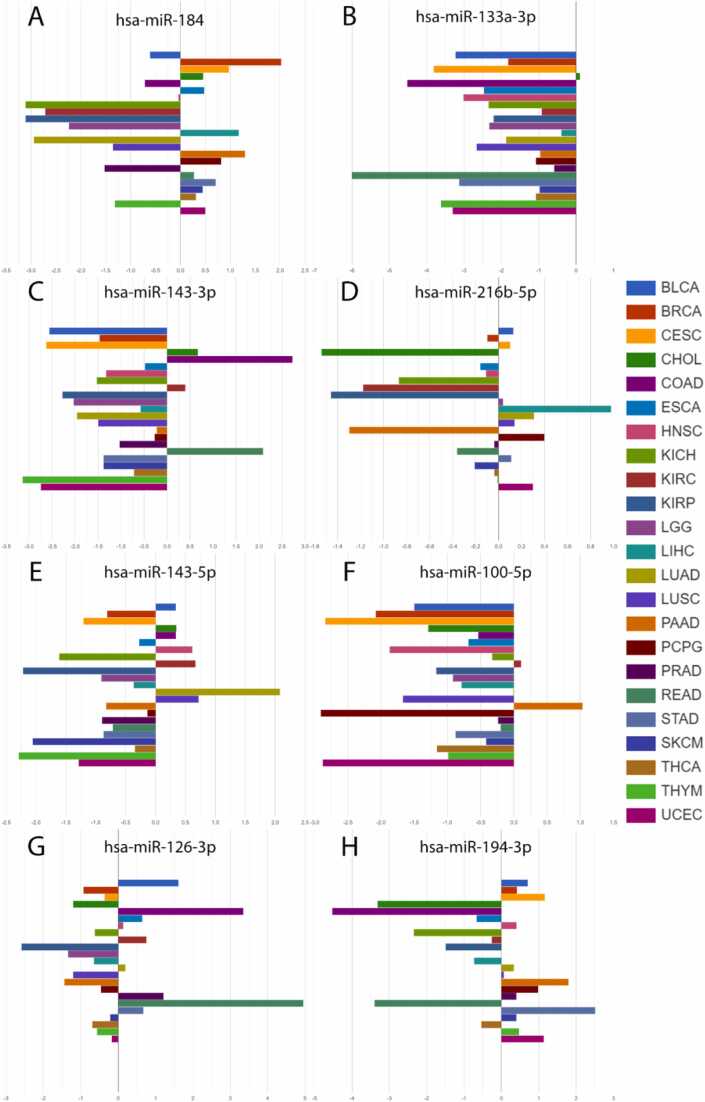
First prioritized miRNAs according to Expression Prioritization Index (EPI) for (A) breast invasive carcinoma (BRCA), (B) cholangiocarcinoma (CHOL), (C) colon adenocarcinoma (COAD), (D) hepatocellular carcinoma (LIHC), (E) lung adenocarcinoma (LUAD), (F) pancreas adenocarcinoma (PAAD), (G) rectum adenocarcinoma (READ), and (H) stomach adenocarcinoma (STAD).

Using AmiCa's "expression by disease" section (Fig. 9), the first-prioritized miRNAs for each cancer were drawn. The first prioritized miRNAs according to EPI were: hsa-miR-184 in BRCA, hsa-miR-133a-3p in CHOL, hsa-miR-143–3p in COAD, hsa-miR-216b–5p in LIHC, hsa-miR-143–5p in LUAD, hsa-miR-100–5p in PAAD, hsa-miR-126–3p in READ and hsa-miR-194–3p in STAD.

## 4 Discussion

We developed AmiCa, a web platform that allows users to analyze and visualize miRNA/gene expression datasets and explore correlations between miRNAs and their target genes. A distinctive feature of our tool is the ability to calculate correlations for both tumor and normal samples, and it includes a ranking system for miRNAs and genes. With AmiCa, researchers can identify prime candidates for targeted hypotheses and design experiments using specific miRNA, gene, or both loci data for advanced research and analysis.

For our first case study demonstrating the capabilities of the AmiCa platform, we selected LGG due to its status as the most common malignant primary brain tumor in adults [19], [20]. Identifying effective biomarkers and new therapeutic targets for diagnosing, treating, and predicting LGG is essential. Notably, genes such as *SPARC* and *VIM* are upregulated in glioblastoma samples [21], [22]. Our demonstration specifically focused on the gene *VIM* and its associated MTIs. The most negatively correlated MTI identified was *VIM*/hsa-miR-124–3p, which is consistent with recent studies confirming the downregulation of hsa-miR-124–3p in gliomas [23].

The second case study was selected to showcase AmiCa's ability to distinguish among different diseases using rank by miRNA/gene. We aimed to identify the most upregulated genes in eight cancer types and compare their expression across other cancers. Notably, distinguishing between similar cancer types that originate in the same organs, such as LIHC and CHOL, as well as COAD and READ, poses a significant challenge based on their expression profiles. Conversely, differences in miRNAs/genes expression are more pronounced among cancers originating from different organs.

We developed a novel index EPI, to identify the most distinctive genetic loci for a specific type of cancer. We prioritized miRNAs and genes with the lowest EPI scores (lower EPI indicating higher priority) and highlighted the top miRNA and gene that are the most distinctive in each of those eight cancer types.

Among these genes, significant evidence shows overexpression of the *GPC3* gene and protein in LIHC [24], [25], [26], and heightened *MUC6* expression was observed in COAD and READ [27]. Additionally, our literature review revealed the upregulation of the *MS4A15* gene has been observed in both STAD [28] and ovarian cancer [29]. *CST1* exhibits overexpression in various cancers, with the highest expression CHOL, and confirmed overexpression in LIHC [30], BRCA [31] and PAAD [32]. In colon cancer, *C6orf15* is highly expressed in tumor tissues, correlating with adverse pathological features and a poor prognosis [33]. The *DKK4* gene has been identified as upregulated in COAD [34]. Many of the loci prioritized within these criteria have not yet been extensively studied in cancer, presenting significant opportunities for further research.

For miRNAs prioritized by the EPI, we observed the upregulation of hsa-miR-100–5p in PAAD [35]. Extensive research has shown that hsa-miR-133a–3p is downregulated in several cancers, including BRCA [36], COAD and READ [37], LIHC [38], STAD [39] and others, aligning with our findings (Fig. 9B). For miRNAs prioritized by each of the criteria we found hsa-miR-374a–3p upregulated in COAD [40], while hsa-miR-552–5p and hsa-miR-26a–5p were overexpressed in COAD and READ [41], [42]. Additionally, hsa-miR-654–5p was found overexpressed in STAD [43] and miRNA hsa-miR-582–3p was also found to be overexpressed in COAD [44].

Future research could explore the differences between primary cancers and their metastases. The Amica tool, as presented here, not only facilitates and complements various studies involving MTI but also empowers researchers to formulate initial hypotheses at the onset of their research endeavors.

Technological advancements in next-generation sequencing have yielded vast genomic data and led to the development of platforms with diverse capabilities. While some platforms offer comprehensive visualization tools for genomic data, others serve as informational hubs for specific miRNAs/genes across various tissues.

AmiCa distinguishes itself by specifically focusing on miRNA-gene target interactions, a feature not comprehensively addressed by other tools. For instance, GEPIA2 [10] excels in gene correlations and disease-specific transcript data but lacks miRNA-target gene pairing. Similarly, dbDEMC [12] provides extensive miRNA expression data across cancers but lacks focus on MTIs. Tissue Atlas [11] offers a comprehensive sncRNA atlas, including miRNA, but limits its correlations among miRNAs in normal tissues. Meanwhile, miTED [45] focuses on tissue origins without exploring detailed MTIs. In contrast, platforms like the Cancer miRNA Census [14] and IMOTA [13] present cancer-related miRNAs and focus on normal tissues, respectively, without providing cancer-specific correlations.

Unlike these platforms, miRTarBase [9] hosts an extensive curated MTI database but is constrained by calculating MTI correlations on paired tumor-normal samples, limiting its wider applicability. In contrast, AmiCa integrates mature miRNA data with gene expression across 32 cancer types, presenting correlations separately for tumor and normal samples where available. This approach allows AmiCa to overcome limitations related to sample availability and ensures biological accuracy by focusing on mature miRNAs, directly involved in posttranscriptional gene regulation. This distinguishes AmiCa from tools like XENA [7], which analyze pre-miRNAs.

AmiCa's unique feature is its ranking system by miRNA/gene, not found in other tools with miRNA/gene expression data. This ranking enhances the platform's utility in oncogenomic research. However, AmiCa faces limitations due to a scarcity of normal tissue samples. Its natural evolution involves expanding the dataset to include more diverse sources and incorporating additional data on normal tissue samples' miRNA/gene expression levels.

In conclusion, AmiCa emerges as a pivotal tool for in-depth exploration of miRNAs and genes within specific cancer contexts. It facilitates comprehensive studies and enables insightful comparisons, significantly contributing to unraveling the complexities of cancer research. This article has highlighted two practical demonstrations of the platform's utility, but it is important to recognize the broader potential that AmiCa offers in advancing cancer research.

## Ethics approval and consent to participate

Not applicable.

## Authors' contributions

D.J.S. and N.H. performed bioinformatics analysis and database construction. D.J.S developed web page. J.P., D.J.S and N.H. conceived and designed the study and wrote the manuscript. All authors edited and approved the final manuscript.

## Funding

This research was funded by the Slovenian Research and Innovation Agency through research core funding No. P3-0054, research core funding No. P4-0133 and through projects J3-3070 and J3-2524.

## CRediT authorship contribution statement

**Nina Hauptman:** Conceptualization, Data curation, Formal analysis, Investigation, Methodology, Project administration, Resources, Visualization, Writing – original draft, Writing – review & editing, Supervision. **Jože Pižem:** Conceptualization, Writing – original draft, Writing – review & editing, Project administration, Supervision. **Daša Jevšinek Skok:** Conceptualization, Data curation, Investigation, Methodology, Resources, Software, Supervision, Visualization, Writing – original draft, Writing – review & editing, Project administration.

## Declaration of Competing Interest

The authors declare no conflict of interest.

## References

[1] Sung H. (2021). Global cancer statistics 2020: GLOBOCAN estimates of incidence and mortality worldwide for 36 cancers in 185 countries. CA Cancer J Clin.

[2] Hasuwa H., Ueda J., Ikawa M., Okabe M. (2013). miR-200b and miR-429 function in mouse ovulation and are essential for female fertility. Science.

[3] Wang Z., Gerstein M., Snyder M. (2009). RNA-Seq: a revolutionary tool for transcriptomics. Nat Rev Genet.

[4] Stark R., Grzelak M., Hadfield J. (2019). RNA sequencing: the teenage years. Nat Rev Genet.

[5] Heath A.P. (2021). The NCI genomic data commons. Nat Genet.

[6] Gao J. (2013). Integrative analysis of complex cancer genomics and clinical profiles using the cBioPortal. Sci Signal.

[7] Goldman M.J. (2020). Visualizing and interpreting cancer genomics data via the Xena platform. Nat Biotechnol.

[8] Kozomara A., Birgaoanu M., Griffiths-Jones S. (2019). miRBase: from microRNA sequences to function. Nucleic Acids Res.

[9] Chou C.H. (2018). miRTarBase update 2018: a resource for experimentally validated microRNA-target interactions. Nucleic Acids Res.

[10] Tang Z., Kang B., Li C., Chen T., Zhang Z. (2019). GEPIA2: an enhanced web server for large-scale expression profiling and interactive analysis. Nucleic Acids Res.

[11] Keller A. (2021). miRNATissueAtlas2: an update to the human miRNA tissue atlas. Nucleic Acids Res.

[12] Xu F. (2022). dbDEMC 3.0: Functional Exploration of Differentially Expressed miRNAs in Cancers of Human and Model Organisms. Genom Proteom Bioinforma.

[13] Palmieri V. (2017). IMOTA: an interactive multi-omics tissue atlas for the analysis of human miRNA–target interactions. Nucleic Acids Res.

[14] Suszynska M. (2024). CMC: Cancer miRNA Census – a list of cancer-related miRNA genes. Nucleic Acids Res.

[15] Xia M., Liu C.J., Zhang Q., Guo A.Y. (2019). GEDS: A Gene Expression Display Server for mRNAs, miRNAs and Proteins. Cells.

[16] Colaprico A. (2016). TCGAbiolinks: an R/Bioconductor package for integrative analysis of TCGA data. Nucleic Acids Res.

[17] Tomasello L., Distefano R., Nigita G., Croce C.M. (2021). The MicroRNA Family Gets Wider: The IsomiRs Classification and Role. Front Cell Dev Biol.

[18] Mount J.& Zumel N. sigr: succinct and correct statistical summaries for reports. 2017.

[19] Gusyatiner O., Hegi M.E. (2018). Glioma epigenetics: From subclassification to novel treatment options. Semin Cancer Biol.

[20] Ostrom Q.T. (2013). CBTRUS statistical report: Primary brain and central nervous system tumors diagnosed in the United States in 2006-2010. Neuro Oncol.

[21] Li Q., Aishwarya S., Li J.-P., Pan D.-X., Shi J.-P. (2022). Gene Expression Profiling of Glioblastoma to Recognize Potential Biomarker Candidates. Front Genet.

[22] Liu Y. (2023). Vimentin promotes glioma progression and maintains glioma cell resistance to oxidative phosphorylation inhibition. Cell Oncol (Dordr ).

[23] Cai S. (2021). miR‑124‑3p inhibits the viability and motility of glioblastoma multiforme by targeting RhoG.. Int J Mol Med.

[24] Sun B. (2017). Significance of Glypican-3 (GPC3) Expression in Hepatocellular Cancer Diagnosis. Med Sci Monit.

[25] Liu X. (2014). Expression of glypican 3 enriches hepatocellular carcinoma development-related genes and associates with carcinogenesis in cirrhotic livers. Carcinogenesis.

[26] Tsuchiya N. (2015). Biomarkers for the early diagnosis of hepatocellular carcinoma. World J Gastroenterol.

[27] Dwertmann Rico S. (2023). Pattern of MUC6 expression across 119 different tumor types: A tissue microarray study on 15 412 tumors. Pathol Int.

[28] Sun L., Zhang Y., Zhang C. (2018). Distinct Expression and Prognostic Value of MS4A in Gastric Cancer. Open Med (Wars).

[29] Fang Y., Yu H., Zhou H. (2022). MS4A15 acts as an oncogene in ovarian cancer through reprogramming energy metabolism. Biochem Biophys Res Commun.

[30] Cui Y. (2019). Upregulation of cystatin SN promotes hepatocellular carcinoma progression and predicts a poor prognosis. J Cell Physiol.

[31] Dai D.N. (2017). Elevated expression of CST1 promotes breast cancer progression and predicts a poor prognosis. J Mol Med (Berl ).

[32] Jiang J., Liu H.L., Liu Z.H., Tan S.W., Wu B. (2015). Identification of cystatin SN as a novel biomarker for pancreatic cancer. Tumour Biol.

[33] Xiong X., Wang S., Gao Z., Ye Y. (2023). C6orf15 acts as a potential novel marker of adverse pathological features and prognosis for colon cancer. Pathol Res Pract.

[34] Pendás-Franco N. (2008). DICKKOPF-4 is induced by TCF/beta-catenin and upregulated in human colon cancer, promotes tumour cell invasion and angiogenesis and is repressed by 1alpha,25-dihydroxyvitamin D3.. Oncogene.

[35] Hara Y. (2023). Dual epigenetic changes in diabetes mellitus-associated pancreatic ductal adenocarcinoma correlate with downregulation of E-cadherin and worsened prognosis. J Pathol Clin Res.

[36] Escuin D. (2021). Circulating microRNAs in Early Breast Cancer Patients and Its Association With Lymph Node Metastases. Front Oncol.

[37] Kong B., Zhao S., Kang X., Wang B. (2021). MicroRNA-133a-3p inhibits cell proliferation, migration and invasion in colorectal cancer by targeting AQP1. Oncol Lett.

[38] Han S. (2020). miR-133a-3p Regulates Hepatocellular Carcinoma Progression Through Targeting CORO1C. Cancer Manag Res.

[39] Zhang X. (2018). Novel role of miR-133a-3p in repressing gastric cancer growth and metastasis via blocking autophagy-mediated glutaminolysis. J Exp Clin Cancer Res.

[40] Li Z., Yao H., Wang S., Li G., Gu X. (2020). CircTADA2A suppresses the progression of colorectal cancer via miR-374a-3p/KLF14 axis. J Exp Clin Cancer Res.

[41] Liu W. (2023). m(6)A‑mediated LINC02038 inhibits colorectal cancer progression via regulation of the FAM172A/PI3K/AKT pathway via competitive binding with miR‑552‑5p. Int J Oncol.

[42] Hishida A. (2022). Investigation of miRNA expression profiles using cohort samples reveals potential early detectability of colorectal cancers by serum miR-26a-5p before clinical diagnosis. Oncol Lett.

[43] Zhou W., Li P., Jin P. (2021). miR-654-5p promotes gastric cancer progression via the GPRIN1/NF-κB pathway. Open Med (Wars).

[44] Bobowicz M. (2016). Prognostic value of 5-microRNA based signature in T2-T3N0 colon cancer. Clin Exp Metastas--.

[45] Kavakiotis I., Alexiou A., Tastsoglou S., Vlachos Ioannis S., Hatzigeorgiou Artemis G. (2021). DIANA-miTED: a microRNA tissue expression database. Nucleic Acids Res.

